# Clonal hematopoiesis of indeterminate potential in high grade B-cell lymphomas: clinicobiological associations and further insight with single-cell multiomics analysis

**DOI:** 10.1038/s41408-026-01541-8

**Published:** 2026-06-20

**Authors:** Caroline Hesselager, Peter Hollander, Emma Pettersson, Gunilla Enblad, Simone Weström, Helena Nord, Arielle R. Munters, Jonas Almlöf, Daniel Eriksson, Panagiotis Baliakas, Rose-Marie Amini

**Affiliations:** 1 Department of Immunology, Genetics and Pathology, Uppsala University, Uppsala, Sweden; 2 Clinical Genomics Uppsala, Science for Life Laboratory, Uppsala University, Uppsala, Sweden

**Keywords:** Cancer, Lymphoma

## To the Editor

Clonal hematopoiesis of indeterminate potential (CHIP) is an age-associated condition linked to enhanced inflammation, cardiovascular disease (CVD) and an inferior outcome in solid malignancies [1, 2]. CHIP is defined as the presence of small circulating clones of hematopoietic cells carrying somatic mutations in genes implicated in myeloid neoplasms (MN) with a variant allele frequency (VAF) ≥2% and no evidence of concurrent MN. The majority of CHIP mutations occur in epigenetic modifier genes such as *DNMT3A* and *TET2*, splicing factors such as *SF3B1* and *SRSF2* or DNA repair genes such as *TP53* and *PPM1D*, which give hematopoietic stem cells a selective advantage by enhancing self-renewal and cell survival [3].

An increased risk of developing lymphomas has been linked to enhanced inflammatory activity contributing to lymphomagenesis [4]. Patients with CHIP also show altered inflammatory responses. CHIP may increase the risk of developing lymphoma [5–7] as has been described in certain T-cell lymphomas, which are sometimes associated with a concurrent MN [8, 9]. We have previously shown that patients with high-grade B-cell lymphomas (HGBCL) and CHIP have an inferior outcome [10] and the association between lymphomas and autoimmune diseases (AID) has previously been explored [4]. We examined the prevalence and clinical impact of CHIP in a large, population-based cohort of HGBCL patients in relation to CVD, AID and secondary malignancies, seeking further plausible explanations for adverse outcomes using single-cell multiomics in a subset of cases.

A total of 176 clinically well-characterized and unselected patients with HGBCLs diagnosed between 2008–2020 were recruited from the Uppsala-Umeå Comprehensive Cancer Consortium (U-CAN) [11]. All patients were diagnosed at the Department of Clinical Pathology, Uppsala University Hospital, according to the World Health Organization (WHO) classification of Tumors of Hematopoietic and Lymphoid Tissues 2008/2016. Clinical data was obtained from medical records. Patients were treated according to Swedish national treatment guidelines, most often with R-CHOP or R-CHOP-like regimens. CVD was defined as hypertension, ischemic heart disease, stroke and other (including heart failure, valvular disease, thromboembolism etc.). AID included rheumatoid arthritis, psoriasis and psoriatic arthritis, Sjögren syndrome, type 1 diabetes mellitus, thyroid disease, inflammatory bowel disease and polymyalgia rheumatica. Further details are shown in Supplementary Table 2.

Peripheral blood samples, collected at diagnosis, were analyzed with targeted next-generation sequencing (NGS) to identify mutations in 33 driver genes recurrently mutated in MN and a variant calling sensitivity of 2% VAF. Further details are described in Supplementary material.

Single-cell analysis of gDNA combined with cell surface protein markers was performed on samples obtained from four patients, using the Tapestri Platform from Mission Bio (San Francisco, California) with version 3 software and reagents. Further details are described in Supplementary material.

Survival curves for overall (OS), progression-free (PFS) and lymphoma-specific survival (LSS) and univariable analyses were performed using the Kaplan-Meier method and compared with the log-rank test. Cox proportional hazards regression was used to estimate adjusted hazard ratios and Student’s *t* test and Wilcoxon rank-sum test for comparison between groups. A *p* < 0.05 was considered statistically significant. OS was measured from date of diagnosis to date of death from any cause. PFS was measured from date of diagnosis to date of relapse or death from any cause. For patients with progressive disease, PFS was zero. LSS was measured from date of diagnosis to date of death from lymphoma; patients who died of other causes were censored. All statistical analyses were performed using RStudio version 2022.07.2.

In 33 patients (19%) pathogenic (P) or likely pathogenic (LP) mutations were detected. *DNMT3A* was the most frequently mutated gene (*n* = 18, in 17 patients), followed by *TET2* (*n* = 7, in 7 patients), *TP53* (*n* = 6, in 5 patients) and *RUNX1* (*n* = 3, in 3 patients). VAF varied from 2–38% (Supplementary Table 3). Variants of uncertain significance (VUS) were observed in 19% (*n* = 33). *BCOR* (*n* = 8, in 4 patients), *KDM6A* (*n* = 6, in 6 patients), *DNMT3A* (*n* = 4, in 4 patients) and *GATA2* (*n* = 4, in 4 patients) were the most frequently mutated genes. VAF varied from 2 to 50% and most of the variants with high VAF suggesting a germline origin were excluded (Supplementary Table 4).

The patients’ clinical characteristics are shown in Supplementary Table 5.

There were no major differences in clinical characteristics, CVD or AID when analyzing the P/LP group or patients with VUS *vs* patients with no variants, and therefore P/LP + VUS were analyzed together (Supplementary Fig. 1 and Supplementary Table 6).

**Fig. 1:**
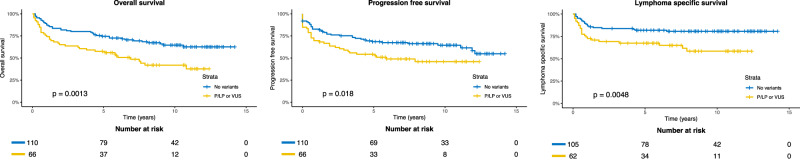
Kaplan-Meier curves of overall, progression-free and lymphoma-specific survival in the whole cohort (*N* = 176) of patients with pathogenic/likely pathogenic (P/LP) or variants of uncertain significance (VUS) compared with patients with no variants.

Patients with P/LP + VUS had a significantly inferior OS (*p* = 0.0013), PFS (*p* = 0.018) and LSS (*p* = 0.0048) (Fig. 1) in univariable analyses. Variables that were statistically significant in univariable analyses for OS were age (*p* < 0.001), aaIPI (*p* = 0.03), CVD (*p* = 0.004) and other malignancies (*p* = 0.016). Variables affecting PFS were aaIPI (*p* = 0.02) and stage (*p* = 0.02). Variables affecting LSS were age (*p* = 0.005), stage (*p* = 0.003) and aaIPI (*p* < 0.001) (Supplementary Table 7).

In multivariable analyses, the presence of CHIP significantly affected OS (hazard ratio (HR) = 1.82, 95% confidence interval (CI): 1.14–2.93, *p* = 0.014). Other parameters that affected survival outcomes were age and disease stage. Patients with P/LP mutations were older (*p* = 0.006), but the association with inferior OS was still significant after adjustment for age, stage and other parameters using a Cox regression model (Supplementary Table 7).

The impact of CHIP was even stronger when primary CNS lymphoma patients were excluded; this entity is regarded as a lymphoma with dismal outcome (Supplementary Table 8 and Supplementary Fig. 1b).

There were no significant differences in the prevalence of CVD or AID between patients with CHIP *vs* patients without variants, nor was any association observed with the presence of another cancer in the multivariable analysis. There were no therapy-related secondary hematologic malignancies recorded in the cohort.

In four patients diagnosed with diffuse large B-cell lymphoma, single-cell multiomics were performed on vital frozen tumor cells. Mutations detected by targeted NGS performed on peripheral blood taken at the time of diagnosis were analyzed in tumor material.

**Fig. 2:**
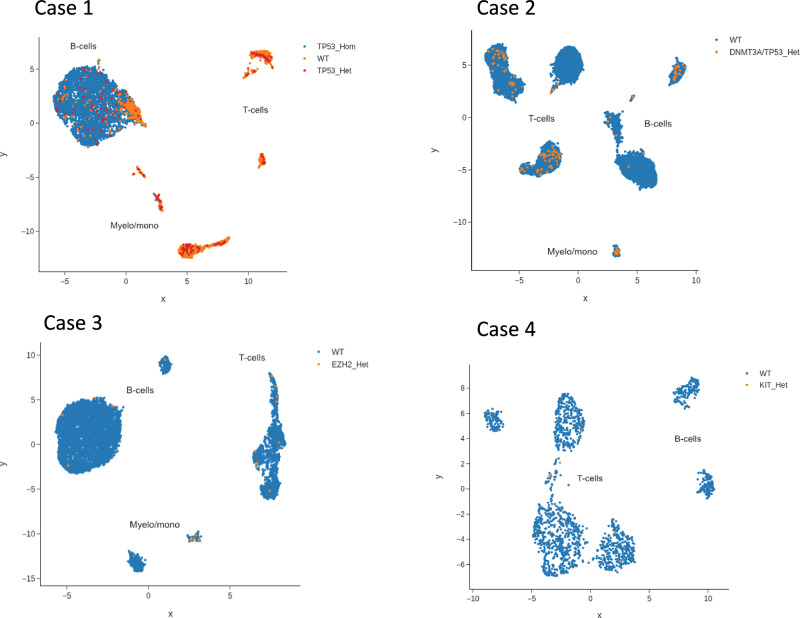
Single-cell multiomics analyses of 4 patients. Case 1–4: UMAP (Uniform Manifold Approximation and Projection) showing gene variants (orange dots, in Case 1 blue and red dots) in clusters of B-cells, T-cells and myeloid cells. Case 1: Targeted NGS in peripheral blood revealed variants in *TP53* with a VAF of 10% and in tumor material the *TP53* variant was homozygous (blue dots) in most of the clustered B-cells and heterozygous (red dots) in subsets of B-cells, T-cells and myeloid cells. WT=wild type (orange dots). Case 2: Targeted NGS of peripheral blood showed variants in *TP53* and *DNMT3A* with VAFs of 2.1% and 2.3%, respectively. In tumor material the mutations coexisted in 0.9% of all cells (orange dots) i.e. in T-cells, myeloid cells, and with varying frequencies in clustered B-cell populations. WT=wild type (blue dots). Case 3: Targeted NGS performed on peripheral blood revealed the presence of an *EZH2* variant with a VAF of 6.4%. In the single-cell analysis performed on tumor material, 0.3% of the cells carried the same variant (orange dots), with the majority being T-cells. WT=wild type (blue dots). Case 4: Targeted NGS in peripheral blood revealed a *KIT* variant with a VAF of 14%. In the single-cell analysis, a small fraction of T-cells harbored the same variant, but the *KIT* variant (orange dots) was not detected in neither B-cells nor myeloid cells. WT=wild type (blue dots).

The results of single-cell multiomics were heterogeneous. In two cases, shared mutations were detected across all cell types, including B-cells, T-cells and myeloid cells. In the remaining two cases, mutations were observed primarily in T-cell populations. Details are shown in Fig. 2, Supplementary Table 9 and Supplementary Fig. 2.

The prevalence of CHIP and the most commonly involved genes (*DNMT3A, TET2* and *TP53)* are in line with previous results [7, 10]. We could not observe any major differences between the P/LP group and cases with no variants regarding any of the clinical or tumor parameters available, although patients with P/LP variants were somewhat older. Patients with VUS also had similar outcomes, indicating that the detection of VUS also may be relevant. The dismal outcome for patients with HGBCL and CHIP could not be explained by an increased prevalence of CVD, since there was no difference in CVD between the different groups. Furthermore, there were no statistically significant associations between CHIP and AID and the prevalence of AID in our cohort was comparable to that reported by others [12]. Except for CVD and AID we did not investigate any inflammatory markers, which is a limitation of the study.

There was no association between the development of secondary malignancies and adverse outcomes. In order to further explore the postulated lymphomagenesis we used single-cell multiomics, hypothesizing that a shared clonal ancestry would render multi-hit genetic aberrations that contribute to poor outcome, which has been observed in some T-prolymphocytic leukemias and indolent B-cell lymphomas [7, 13]. In one patient (case 1), almost all B-cells harbored a homozygous variant of a *TP53* mutation, while a heterozygous *TP53* mutation was detected in T-cells as well as in myeloid cells. This supports the theory of an early hit, which could be consistent with a mutation in an early progenitor cell followed by other genetic aberrations occurring in the B-cell compartment leading to lymphoma development. In case 2, a low frequency of concomitant mutations in *TP53* and *DNMT3A* was observed in most cell types. Thus, a common clonal ancestry for T-, myeloid and malignant B-cells could be observed by a single-cell multiomics approach in two of four investigated cases. In case 3 and 4, however, mutations in *EZH2* and *KIT*, respectively, could mainly be detected in non-malignant T-cells and in a minority of B-cells. This suggests that lymphomagenesis may involve different biological mechanisms. Tumor infiltrating clonal hematopoiesis has been shown to be associated with worse survival in solid cancers [14], and could possibly also have an impact in lymphomas [7]. The small number of cases available for single-cell multiomics is a limitation of our study.

In conclusion, we were able to confirm an adverse outcome for patients with HGBCL and CHIP which was not associated with CVD, AID or secondary malignancies. One potential explanation for the adverse outcome could be an early mutation in a common hematologic ancestral cell, with the acquisition of further genetic aberrations during lymphomagenesis. Another noteworthy observation was the expansion of CHIP-positive immune cells, which might influence the lymphoma tissue microenvironment.

## Supplementary information

Supplementary material

## Data Availability

Data will not be publicly available but shared upon request from the corresponding author.
