# Supplementary material for: Clonal hematopoiesis of indeterminate potential in high grade B-cell lymphomas: clinicobiological associations and further insight with single-cell multiomics analysis
Source: Blood Cancer J. 2026 Jun 20;16(1):98. doi: 10.1038/s41408-026-01541-8 (PMC13283206; doi:10.1038/s41408-026-01541-8)
Supplement: Supplementary file 1 — Supplementary material [file 41408_2026_1541_MOESM1_ESM.docx]

**Supplementary material**

***Patients***

The cohort consisted of 176 patients with HGBCL distributed as follows; Diffuse Large B-cell lymphoma (DLBCL): n=97, primary CNS DLBCL (PCNSL): n=30, Burkitt lymphoma: n=2, High grade B-cell lymphoma with *MYC* and *BCL2* rearrangements: n=3, T-cell/histocyte rich large B-cell lymphoma (THRLBCL): n=8, Primary mediastinal large B-cell lymphoma (PMBL): n=5, B-cell lymphoma unclassifiable, with features intermediate between BL and DLBCL (BCL-U): n=3, Plasmablastic lymphoma: n=1 and HGBCL, not otherwise specified (NOS): n=27. Median age at diagnosis was 65 years (range 17-87) and mean age at diagnosis was 63 years. Median follow up time was 78 months (range 1-161). Age-adjusted International Prognostic Index (aaIPI) was used (scoring one point for each: (1) Ann Arbor stage III-IV; (2) elevated serum lactate dehydrogenase (LDH); (3) and ECOG performance status 2-3), where a score of 0-1 is low risk and score 2-3 is high risk.

***Sequencing***

**NGS library preparation and sequencing using Swift Myeloid panel:**

Genomic DNA was extracted from blood in 103 patients. Genomic DNA was quantified with the Qubit dsDNA BR Assay kit and Qubit 3.0 fluorometer (Thermo Fisher Scientific). Sequencing libraries were prepared from 25 ng input DNA using the Swift-Normalase-Amplicon-Panels (SNAP) protocol with the Myeloid Panel (MY8G1-96) or a custom myeloid panel (Swift Bioscience (now IDT)) according to the manufacturer’s instructions. The myeloid panel consists of 478 unique amplicons (~ 142 base-pairs) covering either hotspots or full coding region of 23 genes that are frequently mutated in myeloid neoplasms (*ASXL1, CALR, CEBPA, CSF3R, DNMT3A, EZH2, FLT3, HRAS, IDH1, IDH2, JAK2, JAK3, KDM6A, KIT, MPL, NPM1, RUNX1, SETBP1, SF3B1, SRSF2, TET2, TP53, U2AF1*). The custom myeloid panel added 353 amplicons to the myeloid panel to cover target regions or genes that are missing in the original panel. The custom panel covers either hotspots or full coding region of 33 genes adding the new targets (*BCOR, BCORL1, CBL, GATA2, KRAS, NRAS, PPM1D, PTPN11, UBA1, ZRSR2*). After PCR clean-up libraries was quantified using the Qubit dsDNA HS Assay kit and fragment size distribution was assessed by an Agilent 4200 Tapestation system (Agilent Technologies) with high sensitivity D1000 Screen Tape. Normalase treated libraries (32 per run) were pooled and denatured. Sequencing was performed on the Illumina NextSeq platform using a MidOutput reagent kit v2 to generate 2x150 bp read lengths as specified by the manufacturer.

The reads were processed using an in-house developed pipeline, available at [https://github.com/clinical-genomics-uppsala/swift_myeloid](https://github.com/clinical-genomics-uppsala/swift_myeloid/), commit 0e9cbfa. Reads were trimmed using fastp (v0.20.1), aligned to the human genome reference GRCh37/hg19 using bwa mem (v0.7.17). The amplicon primer sequences in the aligned reads were trimmed using primerclip (v0.3.8) and variant calling was done using Illumina Pisces (v5.2.11). Pindel (v0.2.5b9) was used to call larger indels over a limited number of genes (*SF3B1, GATA2, KIT, NPM1, FLT3, SRSF2, TP53, PPM1D, CALR, CEBPA, ASXL1, RUNX1*). All calls were normalized and decomposed using vt (v2015.11.0) and annotated using vep (v105). Background allele frequency error rates were estimated based on 190 samples and used to identify artefacts in the sequencing data. Quality values were collected using fastqc (v0.11.9), samtools (v1.15), picard (v2.25.0), and mosdepth (v0.3.2). Variants were annotated using population variation databases and Cosmic (v85) using VEP (v91) and SnpEFF (v4.3) were classified according to an in-house protocol as previously described (1). Most of the variants with high VAF suggesting germline origin were not included. Males with mutations in genes located on chromosome X and >2% VAF were included. Seventy-three patients were analyzed with the Trusight Myeloid Sequencing Panel (Illumina, San Diego, California) covering 47 genes with a cutoff set at 5% VAF as previously reported (2). Comparison of patient characteristics regarding sequencing method are displayed in Supplemental Table 1.

***DNA & protein single-cell multiomics***

**Data generation**

Single-cell analysis of gDNA combined with cell surface protein markers was performed on samples obtained from four patients, using the Tapestri Platform from Mission Bio (San Francisco, California) with version 3 software and reagents. A custom DNA panel consisting of 136 amplicons and covering regions of interest within 25 genes frequently mutated in hematological neoplasms (Mission Bio) and the commercially available TotalSeq™-D Heme Oncology Cocktail (BioLegends, San Diego, California) were used.

Cryopreserved cells were thawed, diluted in RPMI-1640 (Sigma) and washed once with PBS/0,5%BSA. Dead cells were removed using the dead cell removal kit with MS columns (Miltenyi Biotech, Cologne, Germany) and finally cells were resuspended in cell staining buffer (CSB, BioLegends). The cell counts and viability were assessed using a Countess 3 Automated Cell Counter (Thermo Fisher). One million cells at a concentration of 25,000 cells/µl were blocked, stained (Heme Oncology Antibody Cocktail) and washed according to manufacturer’s instructions. After staining, cells were resuspended in Mission Bio’s cell buffer and diluted to a concentration of 3000 cells/µl. Encapsulation, lysis, barcoding and targeted PCR was performed according to the manufacturer’s instructions. PCR products were digested and purified using HighPrep PCR beads (Magbio) followed by library PCR with index primers. After PCR clean-up, each library was quantified using the Qubit dsDNA HS Assay kit (Thermo Fisher) and fragment size distribution was assessed by the Tapestation 4200 (Agilent, San Diego, California) using D1000 Screen Tape. Libraries were pooled (equimolar) followed by sequencing on the NextSeq 2000 using a P3 reagent kit (Illumina, San Diego, California).

**Data analysis**
FASTQ files were analyzed through the Tapestri Pipeline (DNA &Protein v3.4, Mission Bio), which trims adaptor sequences, aligns reads to the human genome (hg19) using BWA aligner, performs barcode correction, assigns sequence reads to unique cell barcodes and performs genotype calling using GATK (v.3.7). Loom and h5 files generated were analyzed using the Mosaic v3.1.1 with the aid of the curated Jupiter notebook downloaded from Mission Bio (<https://missionbio.github.io/mosaic/notebooks/curated_notebooks/dna-protein.html>). Figures produced were also generated using the Jupiter Notebook. Mutations identified in NGS analysis were tracked.

**Supplemental Table 1:** Comparison of patient characteristics regarding different NGS-panels, e.g. Trusight or Swift. Statistical analysis using Wilcoxon rank sum test or Students t-test when marked with *.

|  | **Trusight n=73 (%)** | **Swift n=103 (%)** | **p-value** |
| --- | --- | --- | --- |
| **Age mean** | 62.8 | 63.6 | *0.7 ** |
| **Gender**  Male  Female | 43 (59)  30 (41) | 59 (57)  44 (43) | *0.8* |
| **CHIP (P/LP+VUS)**  P/LP  VUS | 14 (19)  9 (12)  5 (7) | 52 (50)  24 (23)  28 (27) | *<0.001* |
| **CVD** | 41 (56) | 53 (51) | *0.5* |
| **AID** | 9 (12) | 20 (19) | *0.2* |
| **Other malignancy** | 17 (23) | 19 (18) | *0.4* |
| **PCNSL** | 0 | 30 | *<0.001* |
| **Follow up in years** | 8.5 mean  10.1 median (range 0.5-14.2) | 5.6 mean  5.9 median (range 0.1-14.2) | *<0.001 ** |
|  |  |  |  |
|  | n=73 | n=73 (PCNSL excluded) |  |
| **Stage**  1  2  3  4 | 23 (32)  11 (15)  12 (16)  27 (37) | 23 (32)  9 (12)  16 (22)  25 (34) | *0.94* |
| **aaIPI**  0-1  2  3  Unknown | 39 (54)  28 (38)  3 (4)  3 (4) | 46 (63)  23 (32)  3 (4)  1 (1) | *0.87* |
| **B-symptoms**  Yes  No  Unknown | 42 (58)  31 (42) | 26 (36)  46 (63)  1 (1) | *0.01* |

Abbreviations: NGS = next generation sequencing, CHIP=clonal hematopoiesis of indeterminate potential, P/LP = pathogenic/likely pathogenic, VUS = variants of uncertain significance, CVD = Cardiovascular Disease, AID = Autoimmune Disease, aaIPI = age adjusted International Prognostic Index, PCNSL = Primary central nervous system lymphoma

**Supplemental Table 2:** Details of cardiovascular disease (CVD), autoimmune disease (AID) and other malignancies.

| **CVD** | P/LP (n = 19) | | VUS (n = 15) | | No variants (n = 59) | |
| --- | --- | --- | --- | --- | --- | --- |
|  | Diagnosed synchronically or before lymphoma | Diagnosed after lymphoma | Diagnosed synchronically or before lymphoma | Diagnosed after lymphoma | Diagnosed synchronically or before lymphoma | Diagnosed after lymphoma |
| **Total no of events** | 25 | 5 | 13 | 6 | 66 | 26 |
| High blood pressure | 9 | 3 | 8 | 1 | 34 | 4 |
| Ischemic heart disease | 3 | 1 | 1 | 0 | 8 | 2 |
| Stroke | 2 | 1 | 0 | 2 | 4 | 5 |
| Other | 11 | 1 | 4 | 3 | 20 | 16 |
| *-Thrombo*  *embolism* |  |  |  | *2* | *3* | *4* |
| *-Atrial fibrillation* | *4* |  | *2* | *1* | *7* | *6* |
| *-Heart failure* | *3* |  | *1* |  | *2* | *4* |
| *-Valvular disease* |  | *1* | *1* |  | *2* | *1* |
| *-Aortic aneurysm* | *1* |  |  |  | *3* |  |
| *-Cardio myopathy* |  |  |  |  |  | *1* |
| *-Pacemaker* | *3* |  |  |  | *3* |  |

| **AID** | P/LP (n = 7) | | VUS (n = 8) | | No variants (n = 14) | |
| --- | --- | --- | --- | --- | --- | --- |
|  | Diagnosed synchronically or before lymphoma | Diagnosed after lymphoma | Diagnosed synchronically or before lymphoma | Diagnosed after lymphoma | Diagnosed synchronically or before lymphoma | Diagnosed after lymphoma |
| **Total no of events** | 8 | 0 | 9 | 0 | 12 | 2 |
| Thyroid disease | 3 |  | 3 |  | 4 | 2 |
| RA | 1 |  |  |  | 1 |  |
| Sjögren syndrome | 1 |  |  |  | 1 |  |
| IBD | 2 |  | 2 |  | 3 |  |
| Autoimmune hepatitis |  |  | 1 |  |  |  |
| Psoriasis |  |  |  |  | 2 |  |
| Psoriatic arthritis |  |  | 1 |  |  |  |
| DM type 1 |  |  | 1 |  | 1 |  |
| PMR | 1 |  | 1 |  |  |  |

| **Other malignancies** | P/LP (n = 8) | | VUS (n = 3) | | No variants (n = 24) | |
| --- | --- | --- | --- | --- | --- | --- |
|  | Diagnosed synchronically or before lymphoma | Diagnosed after lymphoma | Diagnosed synchronically or before lymphoma | Diagnosed after lymphoma | Diagnosed synchronically or before lymphoma | Diagnosed after lymphoma |
| **Total no of events** | 5 | 4 | 3 | 0 | 22 | 19 |
| Colorectal cancer | 1 |  |  |  | 2 | 7 |
| Esophageal cancer |  |  |  |  |  | 2 |
| Prostate cancer |  |  |  |  | 8 |  |
| Breast cancer | 2 |  |  |  | 1 |  |
| Melanoma |  |  |  |  | 2 |  |
| Squamous cell carcinoma of skin | 1 | 1 |  |  | 1 | 3 |
| Lung cancer |  | 2 |  |  | 1 | 3 |
| Kidney cancer |  |  |  |  | 2 |  |
| Urothelial cancer |  |  |  |  | 1 | 3 |
| Other lymphoma |  | 1 | 1 |  | 3 | 1 |
| Cervical cancer |  |  |  |  | 1 |  |
| Uterine  cancer |  |  | 1 |  |  |  |
| Angiosarcoma |  |  | 1 |  |  |  |
| NET | 1 |  |  |  |  |  |

Abbreviations: P/LP = pathogenic/likely pathogenic, VUS = variants of uncertain significance, RA = rheumatic arthritis, IBD = inflammatory bowel disease, DM = diabetes mellitus, PMR = polymyalgia rheumatica, NET = neuroendocrine tumor

**Supplemental Table 3:** Pathogenic or likely pathogenic variants detected with targeted next generation sequencing (NGS).

| **Patient id no.** | **Gene** | **Chromosome** | **Position** | **Allele frequency** | **Mutation cds** | **Consequence** |
| --- | --- | --- | --- | --- | --- | --- |
| 101 | *DNMT3A* | chr2 | 25457176 | 0,061 | c.2711C>T | missense_variant |
| 102 | *KDM6A* | chrX | 44911002 | 0,030 | c.703G>T | stop_gained |
| 103 | *DNMT3A* | chr2 | 25463585 | 0,342 | c.2096del | frameshift_variant |
| 104 | *DNMT3A* | chr2 | 25463182 | 0,026 | c.2311C>T | stop_gained |
| 105 | *RUNX1* | chr21 | 36164698 | 0,036 | c.1177G>T | stop_gained |
| 106 | *TET2* | chr4 | 106157138 | 0,022 | c.2040del | frameshift_variant |
| 107 | *NPM1* | chr5 | 170837530 | 0,038 | c.847-1G>T | splice_acceptor_variant |
| 107 | *KDM6A* | chrX | 44913201 | 0,023 | c.875+1G>T | splice_donor_variant |
| 108 | *DNMT3A* | chr2 | 25463289 | 0,035 | c.2204A>G | missense_variant |
| 109 | *DNMT3A* | chr2 | 25457243 | 0,032 | c.2644C>T | missense_variant |
| 110 | *TET2* | chr4 | 106156593 | 0,066 | c.1496del | frameshift_variant |
| 111 | *TET2* | chr4 | 106156841 | 0,045 | c.1743dup | frameshift_variant |
| 112 | *SRSF2* | chr17 | 74732959 | 0,246 | c.284C>A | missense_variant |
| 113 | *TP53* | chr17 | 7577099 | 0,023 | c.839G>C | missense_variant |
| 114 | *DNMT3A* | chr2 | 25457242 | 0,376 | c.2645G>A | missense_variant |
| 114 | *DNMT3A* | chr2 | 25469083 | 0,064 | c.1374del | frameshift_variant |
| 115 | *TET2* | chr4 | 106164086 | 0,160 | c.3594+2T>G | splice_donor_variant |
| 116 | *DNMT3A* | chr2 | 25457242 | 0,127 | c.2645G>A | missense_variant |
| 116 | *DNMT3A* | chr2 | 25467408 | 0,026 | c.1667+1G>A | splice_donor_variant |
| 117 | *TP53* | chr17 | 7577539 | 0,103 | c.742C>T | missense_variant |
| 117 | *RUNX1* | chr21 | 36164769 | 0,028 | c.1106C>A | stop_gained |
| 118 | *RUNX1* | chr21 | 36164654 | 0,045 | c.1221C>A | stop_gained |
| 119 | *DNMT3A* | chr2 | 25464494 | 0,023 | c.2017_2018del | frameshift_variant |
| 119 | *TP53* | chr17 | 7578503 | 0,021 | c.427G>A | missense_variant |
| 120 | *DNMT3A* | chr2 | 25468121 | 0,023 | c.1554+1G>A | splice_donor_variant |
| 121 | *DNMT3A* | chr2 | 25457242 | 0,064 | c.2645G>A | missense_variant |
| 122 | *DNMT3A* | chr2 | 25463290 | 0,046 | c.2202del | frameshift_variant |
| 123 | *DNMT3A* | chr2 | 25457242 | 0,021 | c.2645G>A | missense_variant |
| 124 | *TP53* | chr17 | 7578413 | 0,020 | c.517G>T | missense_variant |
| 124 | *ASXL1* | chr20 | 31024986 | 0,021 | c.4471C>T | stop_gained |
| 125 | *KIT* | chr4 | 55594261 | 0.139 | c.1964A>G | missense_variant |
| 126 | TET2 | chr4 | 106180816 | 0.282 | c.3844G>A | missense_variant |
| 127 | *TET2* | chr4 | 106197357 | 0.091 | c.5690T>A | missense variant |
| 128 | *EZH2* | chr7 | 148516718 | 0.064 | c.969del | frameshift variant |
| 129 | *TP53* | chr17 | 7578550 | 0.084 | c.843C>A | missense variant |
| 129 | *TP53* | chr17 | 7577095 | 0.110 | c.380C>T | missense variant |
| 130 | *TET2* | chr4 | 106196833 | 0.074 | c.5166del | frameshift variant |
| 131 | *DNMT3A* | chr2 | 25464531 | 0.243 | c.1982T>C | missense variant |
| 131 | *DNMT3A* | chr2 | 25463295 | 0.065 | c.2198A>G | missense variant |
| 132 | *DNMT3A* | chr2 | 25470535 | 0.064 | p.W313* | stop gained |
| 133 | *DNMT3A* | chr2 | 25463292 | 0.076 | p.E733Lfs*6 | frameshift variant |

**Supplemental Table 4:** Variants of unknown significance detected with targeted next generation sequencing (NGS).

| **Patient id no.** | **Gene** | **Chromosome** | **Position** | **Allele frequency** | **Mutation cds** | **Consequence** |
| --- | --- | --- | --- | --- | --- | --- |
| 134 | *BCOR* | chrX | 39922158 | 1 | c.4014A>C | missense_variant |
| 135 | *GATA2* | chr3 | 128205689 | 0,470 | c.186C>G | missense_variant |
| 136 | *KDM6A* | chrX | 44894207 | 0,025 | c.596C>T | missense_variant |
| 137 | *HRAS* | chr11 | 534257 | 0,13 | c.66G>T | missense_variant |
| 138 | *ASXL1* | chr20 | 31023379 | 0,498 | c.2864G>T | missense_variant |
| 138 | *RUNX1* | chr21 | 36164754 | 0,04 | c.1121C>A | missense_variant |
| 139 | *DNMT3A* | chr2 | 25467472 | 0,028 | c.1604C>T | missense_variant |
| 140 | *JAK2* | chr9 | 5069960 | 0,029 | c.1549G>T | missense_variant |
| 141 | *HRAS* | chr11 | 533827 | 0,096 | c.229G>A | missense_variant |
| 142 | *JAK2* | chr9 | 5069976 | 0,033 | c.1565C>T | missense_variant |
| 143 | *KDM6A* | chrX | 44936004 | 0,038 | c.2921G>T | missense_variant |
| 144 | *HRAS* | chr11 | 534259 | 0,425 | c.64C>T | stop_gained |
| 145 | *GATA2* | chr3 | 128204917 | 0,039 | c.524C>T | missense_variant |
| 146 | *KDM6A* | chrX | 44894194 | 0,023 | c.583G>A | missense_variant |
| 147 | *CEBPA* | chr19 | 33792309 | 0,028 | c.1117C>A | missense_variant |
| 148 | *KDM6A* | chrX | 44919885 | 0,485 | c.1361C>T | missense_variant |
| 149 | *GATA2* | chr3 | 128204963 | 0,025 | c.478A>C | missense_variant |
| 150 | *SF3B1* | chr2 | 198266810 | 0,029 | c.2122G>A | missense_variant |
| 150 | *CEBPA* | chr19 | 33792491 | 0,023 | c.935C>T | missense_variant |
| 151 | *CEBPA* | chr19 | 33792555 | 0,038 | c.871C>A | missense_variant |
| 151 | *KDM6A* | chrX | 44910991 | 0,025 | c.692T>C | missense_variant |
| 152 | *DNMT3A* | chr2 | 25469936 | 0,038 | c.1106T>G | missense_variant |
| 153 | *TET2* | chr4 | 106162532 | 0,084 | c.3447_3461del | inframe_deletion |
| 154 | *ZRSR2* | chrX | 15818074 | 0,058 | c.201G>T | missense_variant & splice_region_variant |
| 154 | *BCOR* | chrX | 39913151 | 0,023 | c.4964C>G | missense_variant |
| 155 | *RUNX1* | chr21 | 36164641 | 0,062 | c.1234G>A | missense_variant |
| 155 | *KDM6A* | chrX | 44911041 | 0,025 | c.742C>A | missense_variant |
| 156 | *ASXL1* | chr20 | 31023666 | 0,470 | c.3151C>T | missense_variant |
| 157 | *BCOR* | chrX | 39922222 | 0,029 | c.3950C>A | missense_variant |
| 158 | *DNMT3A* | chr2 | 25470588 | 0,026 | c.886G>T | missense_variant |
| 159 | *JAK2* | chr9 | 5069949 | 0,034 | c.1538G>T | missense_variant |
| 160 | *DNMT3A* | chr2 | 25464450 | 0,035 | c.2063G>T | missense_variant |
| 161 | *STAG2* | chrX | 123205025 | 0.075 | c.2895G>T | missense_variant |
| 162 | *GATA2* | chr3 | 128205847 | 0.050 | c.360A>T | missense_variant |
| 163 | *STAG2* | chrX | 123205025 | 0.054 | c.2895G>T | missense_variant |
| 164 | *MYD88* | chr3 | 38182638 | 0.483 | c.999G>A | missense_variant |
| 165 | *TET2* | chr4 | 106197548 | 0.49 | c.5881G>C | missense_variant |
| 165 | *ASXL1* | chr20 | 31024002 | 0.48 | c.2357G>A | missense_variant |
| 166 | *BCOR* | chrX | 39923726 | 0,05 | c.3594G>C | missense_variant |
| 166 | *BCOR* | chrX | 39923722 | 0,0507 | c.3598G>C | missense_variant |
| 166 | *BCOR* | chrX | 39923718 | 0,0502 | c.3602G>C | missense_variant |
| 166 | *BCOR* | chrX | 39923727 | 0,051 | c.3593A>T | missense_variant |
| 166 | *BCOR* | chrX | 39923732 | 0,0506 | c.3588A>C | missense_variant |

**Supplemental Table 5:** Patient characteristics. Statistical analysis using Wilcoxon Rank Sum test. Students t-test in variables marked with *

|  | **Total**  **N (%)** | **P/LP**  **N (%)** | **VUS**  **N (%)** | **No variants**  **N (%)** | **P/LP + VUS N (%)** | **P-value**  **P/LP+VUS vs No variants** |
| --- | --- | --- | --- | --- | --- | --- |
| **No of patients** | 176 | 33 (19) | 33 (19) | 110 (62) | 66 (38) |  |
| **Gender** |  |  |  |  |  | *0.94* |
| Male | 102 (58) | 19 (58) | 19 (58) | 64 (58) | 38 (58) |  |
| Female | 74 (42) | 14 (42) | 14 (42) | 46 (42) | 28 (42) |  |
| **Age median** | 65 (range 17-87) | 66.5 (range 47-85) | 65 (range 35-85) | 65 (range 17-87) | 66 (range 35-85) |  |
| **Age mean** | 63.3 | 68.6 | 61.8 | 62.0 | 65.4 | *0.11** |
| **aaIPI** |  |  |  |  |  | *0.13* |
| 0-1 | 99 (56) | 17 (52) | 19 (58) | 64 (58) | 36 (55) |  |
| 2 | 52 (30) | 12 (36) | 7 (21) | 33 (30) | 19 (29) |  |
| 3 | 7 (4) | 2 (6) | 3 (9) | 2 (2) | 5 (7) |  |
| 4 | 0 | 0 | 0 | 0 | 0 |  |
| Unknown | 17 (10) | 2 (6) | 4 (12) | 11 (10) | 6 (9) |  |
| **Stage** |  |  |  |  |  | *0.28* |
| 1 | 45 (26) | 6 (18) | 6 (18) | 34 (32) | 12 (18) |  |
| 2 | 20 (11) | 6 (18) | 3 (9) | 11 (10) | 9 (14) |  |
| 3 | 28 (16) | 6 (18) | 3 (9) | 19 (16) | 9 (14) |  |
| 4 | 52 (30) | 11 (34) | 9 (27) | 32 (29) | 20 (30) |  |
| Primary CNS | 30 (17) | 4 (12) | 12 (36) | 14 (13) | 16 (24) | *0.04* |
| **B-symptoms** |  |  |  |  |  | *0.59* |
| Yes | 68 (39) | 16 (49) | 9 (28) | 43 (39) | 25 (38) |  |
| No | 76 (43) | 13 (39) | 12 (36) | 52 (47) | 25 (38) |  |
| Unknown | 31 (18) | 4 (12) | 12 (36) | 15 (14) | 16 (24) |  |
| **Treatment** |  |  |  |  |  |  |
| RT only | 3 (2) | 0 | 0 | 3 (3) | 0 |  |
| R-CHOP/R-CHOP-like | 139 (79) | 26 (79) | 20 (61) | 93 (85) | 46 (70) |  |
| MPV | 14 (8) | 1 (3) | 7 (21) | 6 (5) | 8 (12) |  |
| Other | 18 (10) | 5 (15) | 6 (18) | 7 (6) | 11 (17) |  |
| aHSCT | 9 (5) | 1 (3) | 5 (15) | 3 (3) | 6 (9) |  |
| None | 2 (1) | 1 (3) | 0 | 1 (1) | 1 (2) |  |
| **Median follow up (months)** | 86 (range 1-172) | 72 (range 1-140) | 63 (range 2-150) | 104 (range 2-172) | 68 (range 1-150) |  |
| **Mean follow up (months)** | 82 | 64 | 65 | 93 | 65 | *<0.001** |
| **High blood pressure** | 60 (34) | 13(39) | 9 (27) | 38 (35) | 22 (33) | *0.87* |
| **Ischemic heart disease** | 15 (9) | 4 (12) | 1 (3) | 10 (9) | 5 (8) | *0.73* |
| **Other cardiovascular conditions** | 41 (23) | 8 (24) | 6 (18) | 27 (25) | 14 (21) | *0.62* |
| **Stroke** | 15 (9) | 4 (12) | 2 (6) | 9 (8) | 6 (9) | *0.84* |
| **Total no of patients with CVD** | 94 (53) | 20 (60) | 15 (45) | 59 (54) | 35 (53) | *0.94* |
| **Other malignancies** | 36 (20) | 9 (27) | 3 (9) | 24 (22) | 12 (18) | *0.56* |
| **Autoimmune diseases** | 29 (16) | 7 (21) | 8 (24) | 14 (13) | 15 (23) | *0.08* |

Abbreviations: DWD = dead with disease. PD = Progressive Disease. RT = Radio Therapy. R-CHO(E)P = rituximab, prednisone, vincristine, cyclophosphamide, doxorubicin, (etoposide). MPV = Metotrexat, Prokarbazin, Vinkristin. aHSCT = autologous hematologic stem cell transplantation. aaIPI = age adjusted International Prognostic Index, CVD = cardiovascular disease.

**Supplemental Table 6:** Univariate comparison between groups. Statistical analysis using Wilcoxon rank sum test. Students t-test when marked with *.

|  | **P/LP vs No variants** | **VUS vs No variants** | **P/LP+VUS vs No variants** | **P/LP vs VUS** |
| --- | --- | --- | --- | --- |
|  | *p-value* | *p-value* | *p-value* | *p-value* |
| **Gender** | 0.95 | 0.95 | 0.98 | 1 |
| **Age (mean)** | **0.005 *** | 0.92 * | 0.11 * | **0.018 *** |
| **aaIPI** | 0.22 | 0.24 | 0.13 | 0.95 |
| **Stage (PCNSL excluded)** | 0.35 | 0.48 | 0.28 | 0.96 |
| **PCNSL** | 0.93 | **0.002** | 0.05 | **0.02** |
| **B-symptoms** | 0.35 | 0.84 | 0.59 | 0.40 |
| **High blood pressure** | 0.61 | 0.44 | 0.87 | 0.30 |
| **Ischemic heart disease** | 0.61 | 0.26 | 0.73 | 0.17 |
| **Other cardiovascular conditions** | 0.97 | 0.45 | 0.62 | 0.56 |
| **Stroke** | 0.49 | 0.69 | 0.84 | 0.40 |
| **Total cardiovascular** | 0.48 | 0.41 | 0.94 | 0.22 |
| **Other malignancy** | 0.52 | 0.10 | 0.57 | 0.06 |
| **Autoimmune disease** | 0.23 | 0.11 | 0.08 | 0.78 |

Abbreviations: P/LP = pathogenic/likely pathogenic, VUS = variants of uncertain significance, aaIPI = age adjusted International Prognostic Index, PCNSL = primary central nervous system lymphoma.

**Supplemental Table 7:** Uni- and multivariable Cox proportional hazard models for overall,

progression-free and lymphoma specific survival (n=176).

|  | **Univariate** | | | **Multivariable** | | |
| --- | --- | --- | --- | --- | --- | --- |
|  | **LSS HR CI (x-y) P** | **OS HR CI (x-y) P** | **PFS HR CI (x-y) P** | **LSS HR CI (x-y) P** | **OS HR CI (x-y) P** | **PFS HR CI (x-y) P** |
| CHIP (VUS included) | 2.27 (1.24, 4.13) **p=0.008** | 2.08 (1.32, 3.29) **p=0.002** | 1.74 (1.01, 2.76) **p=0.019** | 1.85 (0.99, 3.47) p=0.056 | 1.82 (1.14, 2.93) **p=0.014** | 1.53 (0.95, 2.48) p=0.08 |
| Age | 1.04 (1.01,1.07) **p=0.005** | 1.05 (1.03, 1,08) **p<0.001** | 1.01 (0.99, 1.03) p=0.2 | 1.04 (1.01, 1.07) **p=0.003** | 1.05 (1.02, 1.07) **p<0.001** | 1.01 (0.99, 1.03) p=0.4 |
| CVD | 1.24 (0.68, 2.27) p=0.5 | 2.00 (1.23, 3.24) **p=0.004** | 1.07 (0.68, 1.69) p=0.8 | 0.97 (0.52, 1.81) p>0.9 | 1.36 (0.82, 2.25) p=0.2 | 0.97 (0.60, 1.57) p=0.9 |
| AID | 1.24 (0.57, 2.67) p=0.6 | 1.18 (0.65, 2.14) p=0.6 | 1.27 (0.71, 2.27) p=0.4 | 0.95 (0.43, 2.11) p>0.9 | 1.02 (0.54, 1.92) p>0.9 | 1.16 (0.64, 2.11) p=0.6 |
| Other malignancies | 0.88 (0.41, 1.89) p=0.7 | 1.83 (1.12, 3.00) **p=0.016** | 1.08 (0.62, 1.88) p=0.8 | 0.67 (0.28, 1.58) p=0.3 | 1.36 (0.76, 2.41) p=0.3 | 1.17 (0.63, 2.17) p=0.6 |
| aaIPI | 2.12 (1.42, 3.17) **p<0.001** | 1.39 (1.04, 1.84) **p=0.026** | 1.40 (1.05, 1.86) **p=0.022** | Not included | Not included | Not included |
| PCNSL | 2.16 (1.09, 4.30) **p=0.027** | 1.98 (1.16, 3.38) **p=0.012** | 1.96 (1.15, 3.35) **p=0.013** | 1.73 (0.83, 3.62) p=0.2 | 2.22 (1.24, 3.95) **p=0.010** | 1.78 (1.00, 3.14) p=0.06 |
| Stage n=146  (PCNSL excluded) | 1.56 (1.14, 2.14) **p=0.003** | 1.09 (0.92, 1.34) p=0.4 | 1.29 (1.04, 1.61) **p=0.021** | 1.78 (1.27, 2.50) **p<0.001** | 1.28 (1.02, 1.60) **p=0.030** | 1.30 (1.04, 1.63) **p=0.018** |

Abbreviations: LSS = Lymphoma Specific Survival, OS= Overall Survival, PFS= Progression Free Survival, CHIP=clonal hematopoiesis of indeterminate potential, VUS = variants of uncertain significance, CVD = Cardiovascular Disease, AID = Autoimmune Disease, aaIPI = age adjusted International Prognostic Index, PCNSL = Primary central nervous system lymphoma

**Supplemental Table 8:** Uni- and multivariable Cox proportional hazard models for overall, progression-free and lymphoma specific survival, PCNSL excluded (n=146).

|  | **Univariate** | | | **Multivariable** | | |
| --- | --- | --- | --- | --- | --- | --- |
|  | **LSS HR CI (x-y) P** | **OS HR CI (x-y) P** | **PFS HR CI (x-y) P** | **LSS HR CI (x-y) P** | **OS HR CI (x-y) P** | **PFS HR CI (x-y) P** |
| CHIP (incl VUS) | 2.98  (1.48, 6.00) **p=0.002** | 2.29  (1.36, 3.86) **p=0.002** | 2.02  (1.19, 3.44) **p=0.009** | 2.77  (1.36, 5.66) **p=0.005** | 2.29  (1.34, 3.92) **p=0.003** | 1.93  (1.13, 3.29) **p=0.018** |
| Age | 1.04  (1.01, 1.08) **p=0.003** | 1.06  (1.04, 1,09) **p<0.001** | 1.01  (0.99, 1.03) p=0.2 | 1.06  (1.02, 1.10) **p=0.001** | 1.06  (1.03, 1.09) **p<0.001** | 1.01  (0.99, 1.03) p=0.3 |
| CVD | 1.40  (0.69, 2.84) p=0.3 | 2.79  (1.55, 5.05) **p<0.001** | 1.16  (0.68, 1.97) p=0.6 | 1.33  (0.63, 2.81) p=0.5 | 1.85  (0.98, 3.48) p=0.050 | 1.09  (0.62, 1.94) p=0.8 |
| AID | 1.42  (0.58, 3.44) p=0.5 | 1.02  (0.48, 2.14) p>0.9 | 1.35  (0.68, 2.67) p=0.4 | 0.92  (0.36, 2.35) p=0.9 | 0.75  (0.34, 1.68) p=0.5 | 1.17  (0.57, 2.41) p=0.7 |
| aaIPI | 2.26  (1.46, 3.49) **p<0.001** | 1.43  (1.06, 1.94) **p=0.020** | 1.44  (1.07, 1.94) **p=0.017** | Not included | Not included | Not included |
| Stage | 1.56  (1.14, 2.14) **p=0.003** | 1.09  (0.89, 1.34) p=0.4 | 1.29  (1.04, 1.61) **p=0.021** | 1.78  (1.27, 2.50) **p<0.001** | 1.28  (1.02, 1.60) **p=0.030** | 1.30  (1.04, 1.63) **p=0.018** |
| Other malignancy | 1.03  (0.46. 2.30)  p>0.9 | 2.25  (1.31, 3.85) **p=0.003** | 1.22  (0.67, 2.21) p=0.5 | 0.58  (0.23, 1.44) p=0.2 | 1.15  (0.62. 2.15) p=0.7 | 1.10  (0.57. 2.12) p=0.8 |

Abbreviations: LSS = Lymphoma Specific Survival, OS= Overall Survival, PFS= Progression Free Survival, CHIP=clonal hematopoiesis of indeterminate potential, VUS = variants of uncertain significance, CVD = Cardiovascular Disease, AID = Autoimmune Disease, aaIPI = age adjusted International Prognostic Index, PCNSL = Primary central nervous system lymphoma

**Supplemental Table 9:** Case 1-4 included in the single-cell multiomics analysis.

|  | **Age** | **Stage** | **Gene** | **NGS VAF** | **Single-cell VAF** | **Cell type** | **Initial treatment** | **Outcome** |
| --- | --- | --- | --- | --- | --- | --- | --- | --- |
| Case 1 | 59 | 4 | *TP53* | 10% | 57% homozygous | B-cells | R-CHOP and intrathecal methotrexate | DWD |
|  |  |  |  |  | 3.1% heterozygous | B-cells, T-cells and myeloid cells |  |  |
| Case 2 | 67 | 3 | *TP53* | 2.1%, | 0.9% *TP53/DNMT3A* | B-cells,  T-cells and myeloid cells | R-CHOP | ADF |
|  |  |  | *DNMT3A* | 2.3% |  |  |  |  |
| Case 3 | 65 | 2 | *EZH2* | 6.4% | 0.3% | T-cells | R-CHOP | ADF |
| Case 4 | 82 | 1 | *KIT* | 14% | 0.4% | T-cells | R-CHOP | DDF |

Abbreviations: NGS=next generation sequencing, VAF=variant allele frequency, ADF=alive disease free, DWD=dead with disease, DDF=dead disease free. R-CHOP= rituximab, prednisone, vincristine, cyclophosphamide, doxorubicin

**Supplemental Figure 1a:** Kaplan-Meier curves of overall, progression free and lymphoma specific survival. Pathogenic/likely pathogenic (P/LP) vs No variants and variants of uncertain significance (VUS) vs No variants respectively.

P/LP: VUS:

**Supplemental Figure 1b**: Kaplan-Meier curves of overall, progression free and lymphoma specific survival. P/LP or VUS vs No variants. PCNSL excluded.

**Supplemental Figure 2**

DNA and protein heatmaps with clustering of cells based on protein expression and cluster tables disclosing variant fractions in different cell clusters in Case 1-4.

**Case 1**

**
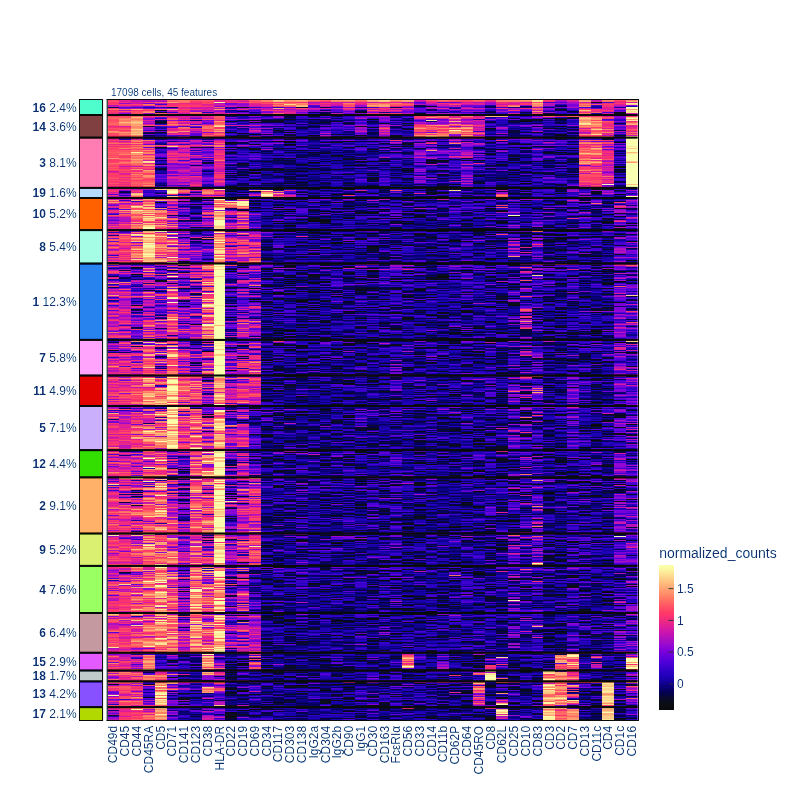
**

B-cells

Myeloid cells

T-cells

**
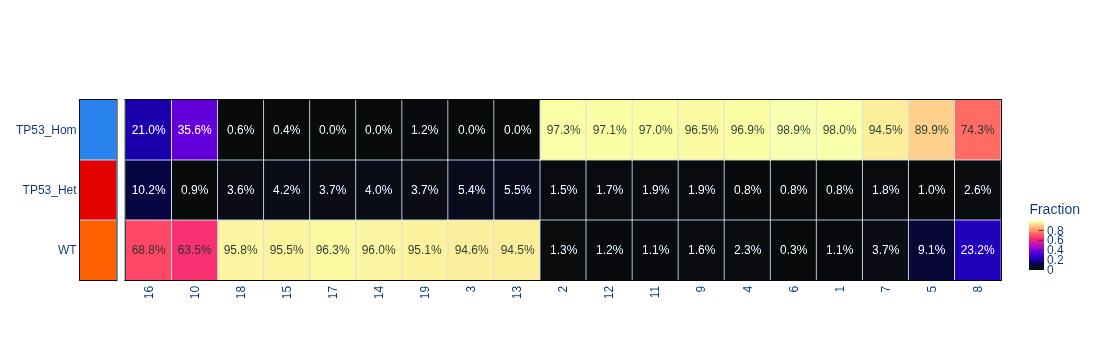
**

**Case 2**

T-cells

B-cells

Myeloid cells

**
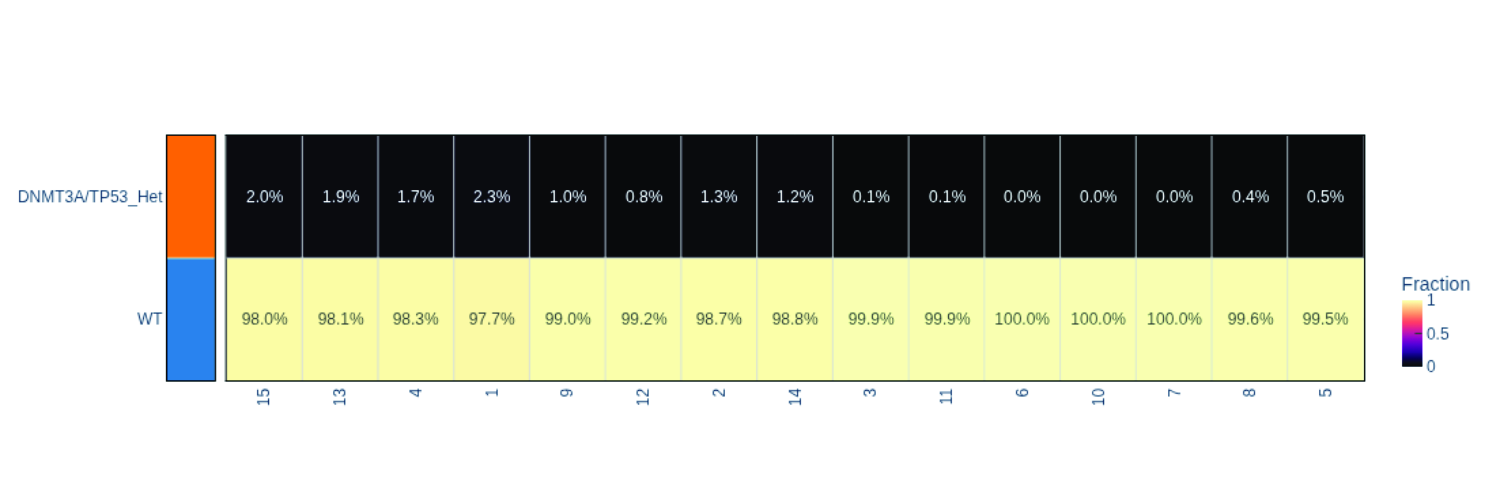
**

**
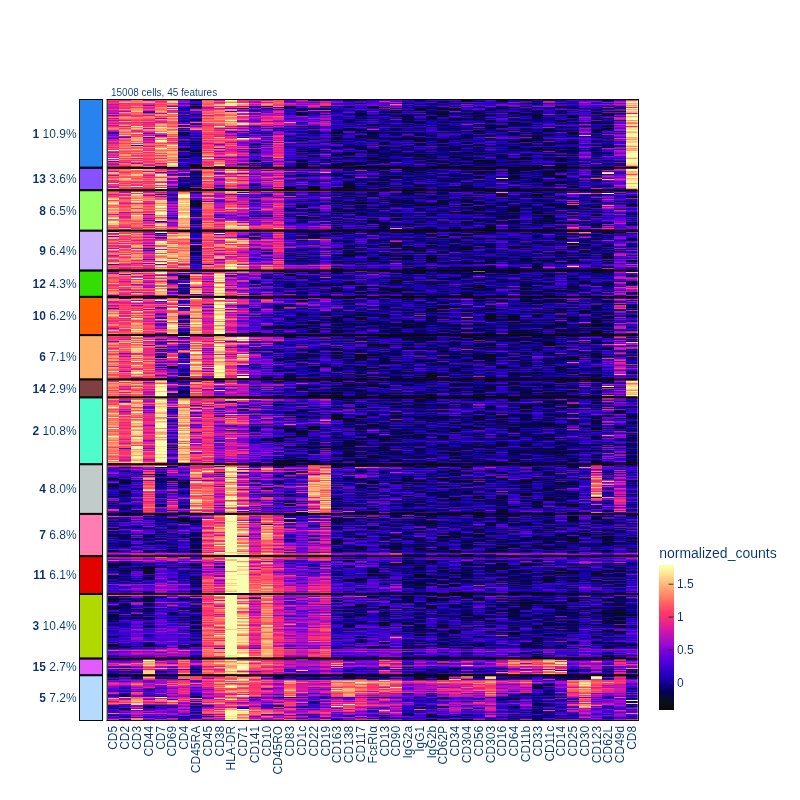
**

**Case 3**

Myeloid cells

T-cells

B-cells

**Case 4**

T-cells

B-cells

Myeloid

cells

**References:**

fastp:

Shifu Chen, Yanqing Zhou, Yaru Chen, Jia Gu, fastp: an ultra-fast all-in-one FASTQ preprocessor, Bioinformatics, Volume 34, Issue 17, September 2018, Pages i884–i890, <https://doi.org/10.1093/bioinformatics/bty560>

fastqc:

Andrews, S. (2010). FastQC:  A Quality Control Tool for High Throughput Sequence Data [Online]. Available online at: <http://www.bioinformatics.babraham.ac.uk/projects/fastqc/>

samtools:
Heng Li, Bob Handsaker, Alec Wysoker, Tim Fennell, Jue Ruan, Nils Homer, Gabor Marth, Goncalo Abecasis, Richard Durbin, 1000 Genome Project Data Processing Subgroup, The Sequence Alignment/Map format and SAMtools, Bioinformatics, Volume 25, Issue 16, August 2009, Pages 2078–2079, <https://doi.org/10.1093/bioinformatics/btp352>

picard:

@misc{Picard2019toolkit,

title = {Picard toolkit},

year = {2019},

publisher = {Broad Institute},

journal = {Broad Institute, GitHub repository},

howpublished = {\url{https://broadinstitute.github.io/picard/}}

}

mosdepth:

Pedersen BS, Quinlan AR. Mosdepth: quick coverage calculation for genomes and exomes. Bioinformatics (Oxford, England). 2018 Mar;34(5):867-868. DOI: 10.1093/bioinformatics/btx699. PMID: 29096012; PMCID: PMC6030888.

Vep:

McLaren, W., Gil, L., Hunt, S.E. *et al.* The Ensembl Variant Effect Predictor. *Genome Biol* **17**, 122 (2016). https://doi.org/10.1186/s13059-016-0974-4

vt:
Adrian Tan, Gonçalo R. Abecasis, Hyun Min Kang, Unified representation of genetic variants, Bioinformatics, Volume 31, Issue 13, July 2015, Pages 2202–2204, <https://doi.org/10.1093/bioinformatics/btv112>

bwa mem:

Li H. (2013) Aligning sequence reads, clone sequences and assembly contigs with BWA-MEM. [arXiv:1303.3997v2](http://arxiv.org/abs/1303.3997) [q-bio.GN]

pindel:

Ye K, Schulz MH, Long Q, Apweiler R, Ning Z. Pindel: a pattern growth approach to detect break points of large deletions and medium sized insertions from paired-end short reads. Bioinformatics. 2009 Nov 1;25(21):2865-71. doi: 10.1093/bioinformatics/btp394. Epub 2009 Jun 26. PMID: 19561018; PMCID: PMC2781750.

Primerclip:

[*https://github.com/swiftbiosciences/primerclip*](https://github.com/swiftbiosciences/primerclip)

pisces:

Tamsen Dunn, Gwenn Berry, Dorothea Emig-Agius, Yu Jiang, Serena Lei, Anita Iyer, Nitin Udar, Han-Yu Chuang, Jeff Hegarty, Michael Dickover, Brandy Klotzle, Justin Robbins, Marina Bibikova, Marc Peeters, Michael Strömberg, Pisces: an accurate and versatile variant caller for somatic and germline next-generation sequencing data, Bioinformatics, Volume 35, Issue 9, May 2019, Pages 1579–1581, <https://doi.org/10.1093/bioinformatics/bty849>

1. Baliakas P, Tesi B, Cammenga J, Stray-Pedersen A, Jahnukainen K, Andersen MK, et al. How to manage patients with germline DDX41 variants: Recommendations from the Nordic working group on germline predisposition for myeloid neoplasms. Hemasphere. 2024;8(8):e145.

2. Amini RM, Ljungstrom V, Abdulla M, Cavelier L, Pandzic T, Hollander P, et al. Clonal hematopoiesis in patients with high-grade B-cell lymphoma is associated with inferior outcome. Am J Hematol. 2020.
